# The porphyrin center as a regulator for metal–ligand covalency and π hybridization in the entire molecule†

**DOI:** 10.1039/d1cp03944j

**Published:** 2021-10-29

**Authors:** Robby Büchner, Mattis Fondell, Robert Haverkamp, Annette Pietzsch, Vinícius Vaz da Cruz, Alexander Föhlisch

**Affiliations:** Institute of Physics and Astronomy, University of Potsdam Karl-Liebknecht-Str. 24-25 14476 Potsdam Germany rbuechner@uni-potsdam.de +49 30 806213967; Institute for Methods and Instrumentation for Synchrotron Radiation Research, Helmholtz-Zentrum Berlin für Materialien und Energie Albert-Einstein-Str. 15 12489 Berlin Germany vinicius.vaz_da_cruz@helmholtz-berlin.de +49 30 806214987

## Abstract

The central moiety of porphyrins is shown to control the charge state of the inner complex and links it by covalent interaction to the peripheral substituents. This link, which enables the versatile functions of porphyrins, is not picked up in the established, reduced four orbital picture [Gouterman, *J. Mol. Spectrosc.*, 1961, **6**, 138]. X-ray absorption spectroscopy at the N K-edge with density functional theory approaches gives access to the full electronic structure, in particular the π* manifold beyond the Gouterman orbitals. Systematic variation of the central moiety highlights two linked, governing trends: The ionicity of the porphyrin center increases from the aminic N–H to N–Cu to N–Zn to N–Mg to the iminic N:. At the same time covalency with peripheral substituents increases and compensates the buildup of high charge density at the coordinated nitrogen sites.

## Abstract 2

The porphyrin center is shown to control both the covalency of the central complex and π hybridization with peripheral substituents.

## Graphical abstract

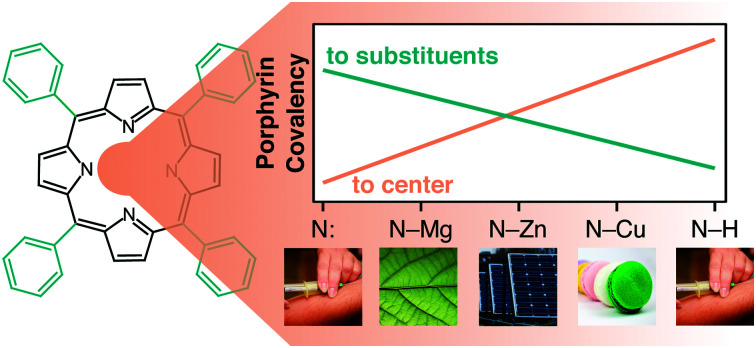

## 1 Introduction

While the class of porphyrins and derivatives is of versatile use and of significant importance both in nature and technology, each function is fulfilled by a porphyrin with particular constitution: protoporphyrin IX, for instance, contains a center of two hydrogen atoms, which are replaced by magnesium in plants to form chlorophyll or iron in vertebrates to form haeme. Upon iron deficiency zinc protoporphyrin is synthesized by the body, instead, leading to anemia.^1^ If the human porphyrin metabolism is substantially impaired, protoporphyrin IX accumulates in the body causing photoallergy and hepatic disease.^2^ Analogously to the necessity for an iron center in heame, the magnesium center of chlorophyll is essential for light-harvesting in plants. For the technical imitation of photosynthesis, however, copper^3^ or zinc^4^ are used as central atoms, since isolated chlorophyll easily disintegrates.^5^ In contrast, the high stability of copper chlorophyllins allows their application in the food industry,^6^ catalysis,^7^ and medicine.^8^ The use of chlorophyllins is also supported by their high aqueous solubility. The ability of porphyrins and derivatives to dissolve in polar solvents, bind to synthetic surfaces or proteins in a specific geometric arrangement are governed by the peripheral substituents which differ for each before mentioned application in addition to the central moiety.

**Fig. 1:**
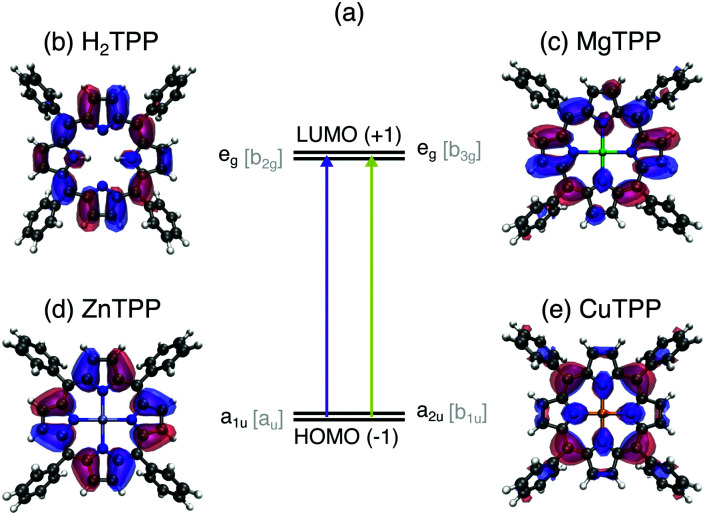
Frontier orbitals of the series of tetraphenylporphyrin (TPP) cores in relation to optical properties: (a) optical transitions between the near-degenerate LUMO/LUMO+1 (b and c) and HOMO−1/HOMO (d and e) orbitals of all porphyrins give rise to the common UV/VIS absorption bands – the foundation for the Gouterman model. Illustrations of these Gouterman orbitals (named after their symmetry in the *D*_4h_ [*D*_2h_] point group) in the TPPs: (b) e_g_ [b_2g_] of H_2_TPP, (c) e_g_ [b_3g_] of MgTTP, (d) a_1u_ [a_u_] of ZnTTP, (e) a_2u_ [b_1u_] of CuTTP.

Considering the vast variety of porphyrins, it is remarkable that all of them show similar optical properties (see UV/VIS spectra in the ESI†). The strong UV (Soret or B band) and weaker visible (Q) bands of all porphyrins have been explained by π–π* transitions from the two highest occupied molecular orbitals (HOMO, HOMO−1 – see Fig. 1a) to the two lowest unoccupied molecular orbitals (LUMO, LUMO+1) by Gouterman in Gouterman 1961.^9^ This four orbital model explains the intensity differences and the splitting of the visible bands in H_2_TPP by assuming the HOMO(+1) to be accidentally degenerate. This condition can be weakened by mixing of metal p_*z*_ atomic orbitals with the HOMO or electron donating/accepting side chains. However, optical spectroscopy of porphyrins is rather insensitive to electronic structure changes upon peripheral substitution or exchange of the central moiety. Since past experimental electronic structure studies were focused on the Gouterman orbitals, theoretical approaches lack precision on the π manifold^10^ and the description of differences between the porphyrin species.

In this work, we investigate the influence of a 2H^+^ (Fig. 1b), Mg^2+^ (Fig. 1c), Zn^2+^ (Fig. 1d), and Cu^2+^ center (Fig. 1e) on the electronic structure of tetraphenylporphyrins (TPPs) and their carboxylated derivatives (TCPPs) in comparison to the parent, unsubstituted porphyrins (Ps). For this purpose, we exploit the element selectivity of Near-Edge X-ray Absorption Spectroscopy (NEXAFS) at the nitrogen atoms, which link the porphyrin macrocycle to the central moiety. To uncover the nature of the porphyrin transition beyond the Gouterman orbitals, density functional theory calculations are carried out for a range of functionals both with the transition potential (TP-DFT) and time dependent approach (TD-DFT). Thereby we establish the porphyrin center as a regulator for the hybridization in the entire molecule controlling both the ionicity of the metal–ligand bond and the π covalency of the macrocycle with peripheral substituents.

## 2 Methods

### 2.1 Experimental details

For the experimental investigation 5,10,15,20-tetrakis(4-carboxyphenyl)porphyrins (TCPPs) have been selected, since the carboxyl groups in the *para* position of each phenyl substituent drastically increase the solubility of the *per se* mostly nonpolar porphyrins in aqueous solution. These TPP derivatives were synthesized by Por-Lab (Scharbeutz, Germany) with a minimum purity of 95%. To ensure a maximum concentration of the investigated solutions, the powder samples were dissolved in a 0.01 N NaOH solution. After one hour of ultrasonic treatment, they were filtered through a 25–50 μm filter. The final concentrations were determined by UV/VIS absorption measurements (Shimadzu UV-2700) yielding approximately 3.0 mM for H_2_TCPP, 3.6 mM for MgTCPP, 4.6 mM for ZnTCPP, and 2.9 mM for CuTPP.

In order to directly access the absorbance of soft X-rays by transmission measurements, the thickness of the sample needs to be in the order of a few μm. Such a condition is provided by the nmTransmission NEXAFS endstation,^11^ where the liquid is pushed into the vacuum chamber through two 30 μm sized nozzles. Upon collision, the two jets form a liquid sheet, whose thickness can be adjusted to suit the penetration depth of soft X-rays. In the present study, a flow rate of 1.6 ml min^−1^ was used resulting in thicknesses of approximately 4 μm. The continuous replenishment of the solutions also prevents radiation induced sample damage.

The experiment was prepared at beamline UE49-SGM^12^ (Bessy II, Helmholtz-Zentrum Berlin) with the EDAX endstation^13^ and finally conducted at the neighboring beamline UE52-SGM.^14^ The spectra were on average acquired for 22 s for each 0.05 eV step. The bandwidth of the incoming X-rays was 0.13 eV.

The resulting spectra were calibrated by the NEXAFS signature of co-dissolved N_2_ at 400.84 eV.^15,16^ The solvent background including the N_2_ features has been measured separately, fitted and subtracted from the sample spectra yielding the pure NEXAFS of aqueous carboxylated TPPs.

### 2.2 Computational details

Both 5,10,15,20-tetraphenylporphyrins (TPPs, as shown in Fig. 1) and the parent Ps, in which four hydrogen atoms replace the phenyl groups, were investigated by electronic structure calculations to distinguish the effects of substitution and different central atoms.

All geometry optimizations and TD-DFT calculations were carried out with the Orca package.^17^ Molecular geometries were optimized with the PBE0^18^ functional and def2-TZVP(-f)^19^ basis set without symmetry constraints to account for the tilt of the phenyl substituents (see Section 3.3). The RIJCOSX approximation^20^ was used with the def2/J^21^ auxiliary basis set. Becke–Johnson damping^22,23^ was used for dispersion correction. For the TD-DFT X-ray absorption spectrum calculations the def2-TZVP basis was used for all atoms except for the central metal atom and the nitrogen atoms, on which the diffuse def2-TZVPD basis set was adopted. In the calculations, the near-degenerate core-orbitals were localized and the multiplicity of the states involved has been restricted to doublet–doublet (CuTPP) and singlet–singlet excitations (H_2_TPP, MgTPP, ZnTPP). For the simulation of the aqueous environment (resulting in the spectra shown in Fig. 4a) the conductor-like polarizable continuum model^24^ (CPCM) has been used both in the geometry optimization and TD-DFT calculations.

The choice of the density functional kernel for porphyrins poses a difficult task, specially concerning the X-ray absorption spectrum simulations. Although, the lowest π* resonance is typically well described by global hybrids^25^ and consequently all functionals tested here were able to correctly describe the transition from the N 1s orbital to the e_g_ Gouterman orbitals. For large conjugated systems, such as porphyrins, additional π* resonances occur below the ionization threshold, which might not be properly described by a functional with large exact exchange fraction. On the one hand, the description of higher lying π* orbitals is worse for global hybrids with a high fraction of exact exchange (see DFT benchmark in the ESI†). On the other hand, the description of metal–ligand charge transfer states requires hybrid functionals, including large fractions of Hartree–Fock exchange (HFX).^10,26,27^ Since no clear single compromise could be reached we have used the BLYP^28,29^ functional for analyzing the transitions of π character, while the transitions affected by metal–ligand interactions were studied by the BHandHLYP^30^ functional.

To assess the role of core–hole relaxation, additional spectral calculations based on the transition potential (TP-DFT) method were carried out. The PSIXAS^31^ plugin for PSI4^32^ was used and the same basis sets used for the TD-DFT calculations described above to enable comparability. The excited state was calculated in the presence of a half core hole.

The resulting transition energies were shifted according to the first experimental resonance of MgTCPP (−1.96 eV for TP-DFT BLYP, +20.29 eV for TD-DFT BLYP, +1.26 eV for TD-DFT BHandHLYP) or the respective resonance for which interspecies shifts are plotted (−0.21 eV for the second resonance in Fig. 4b). The final spectra were obtained by convolution with a Voigt profile of 0.13 eV^33^ (Lorentzian FWHM) and 0.20 eV (Gaussian FWHM) broadening.

Isosurface plots are displayed for the virtual Kohn–Sham orbitals obtained by ground state calculation of CuTPP with an isovalue of 0.02. The orbitals for the remaining systems are visually analogous.

## 3 Results and discussion

**Fig. 2:**
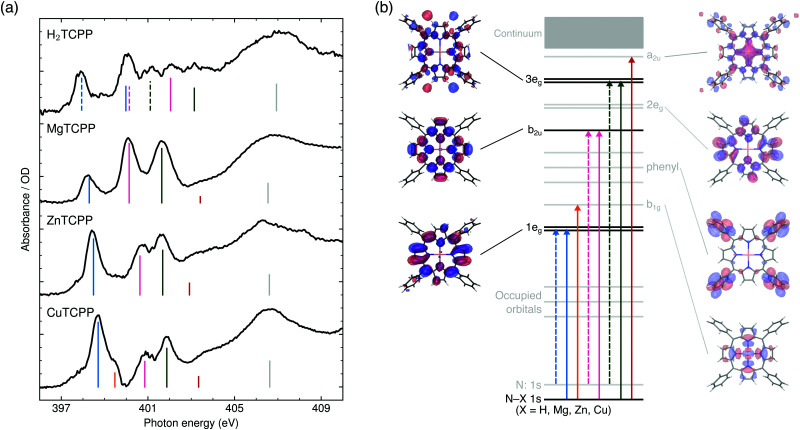
(a) Experimental N K-edge NEXAFS of carboxylated TPPs. The interpretation of the main resonances (see ESI† for spectral decomposition) is indicated by the color of the dashed (excitation from an iminic nitrogen atom) and solid vertical lines (other nitrogen environments): 1e_g_ (blue), b_1g_ (orange), b_2u_ (pink), 3e_g_ (dark green), a_2u_ (red), shape resonance (gray). (b) Schematic representation of the transitions with equivalent line styles. The orbitals which are involved in the NEXAFS of all investigated species are illustrated in opaque colors.

In Fig. 2(a) the N K-edge NEXAFS of carboxylated TPPs with 2H^+^, Mg^2+^, Zn^2+^, and Cu^2+^ as central moiety are shown. It can be seen that three resonances appear for each nitrogen atom in a specific chemical environment. The decomposition of the H_2_TPP spectrum in transitions of the aminic (N–H, solid lines) and iminic nitrogen atoms (N:, dashed lines) was firstly proposed by Narioka *et al.*^34^ and is confirmed by our calculations. The interpretation of the experimental features, which will be discussed below, is summarized in Fig. 2(b). In short, the 1e_g_ (blue), b_2u_ (pink), and 3e_g_ (dark green) resonances are observed for each sample and nitrogen site. To enable comparability to past studies with symmetry restriction or different substituents, (instead of N(1s) → HOMO, N(1s) →…) the transitions are classified by the symmetry of the vacant orbital in the idealized *D*_4h_ point group of the porphyrin macrocycle and numbered by the respective occurrence above the Fermi level. The nomenclature for the Gouterman orbitals in the reduced idealized symmetry of H_2_TPP (*D*_2h_) is given in Fig. 1.

It should be noted, that the electronic transitions observed in the X-ray absorption spectrum at typical light element K-edges are dressed by sizable Franck–Condon progressions, which combined with the electronic transition dipole moment determine the intensity ratios.^35–37^ The role of vibrational excitations is also evidenced by the large width of the observed near-edge resonances.

**Fig. 3:**
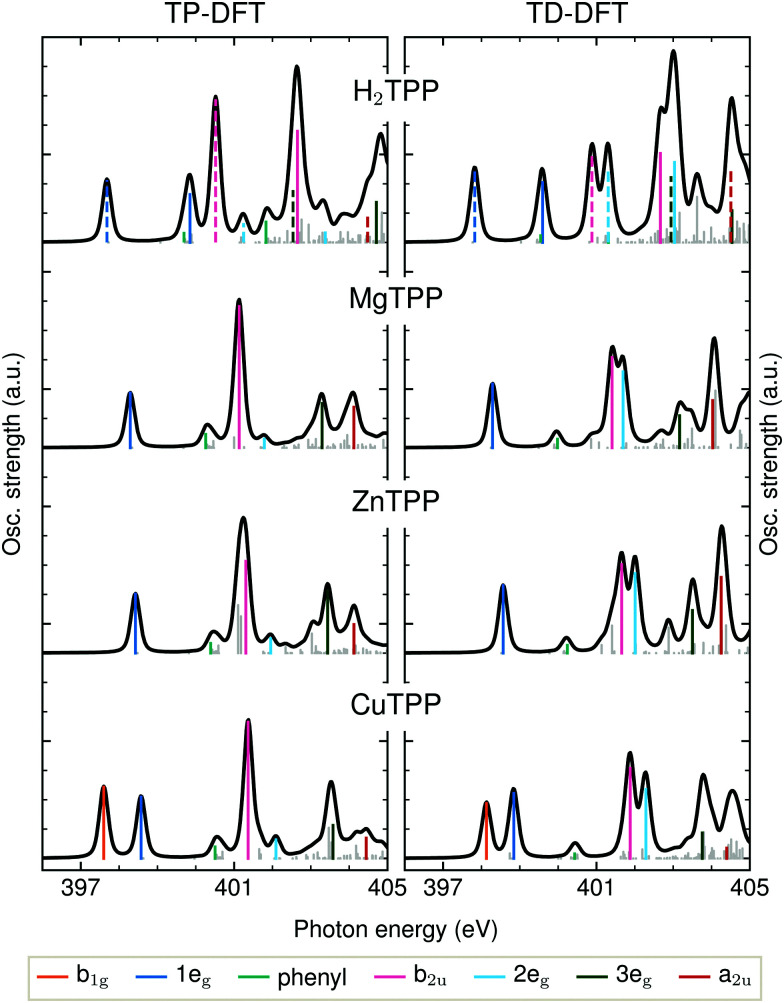
Transition potential and time dependent DFT calculations of the TPP N K-edge with the BLYP functional. Dashed transition result from the excitation of a core electron of an iminic nitrogen atom.

**Fig. 4:**
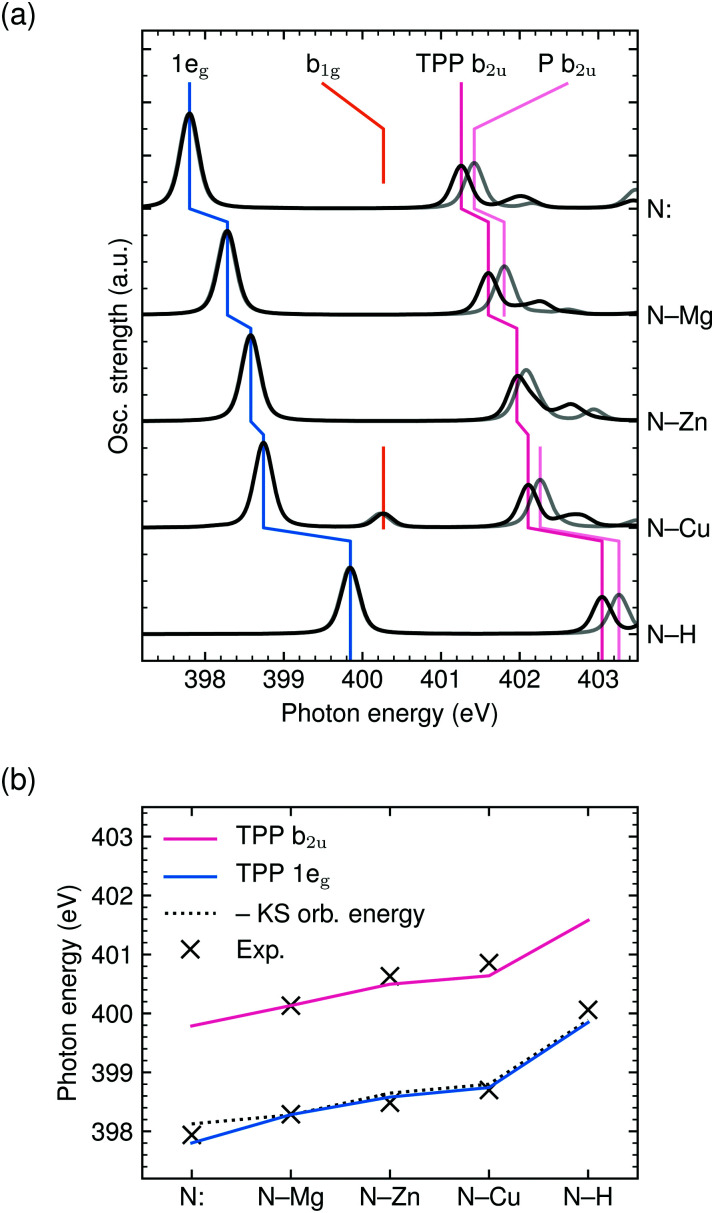
Blue-shift of the 1e_g_ and b_2u_ transition energies from iminic nitrogen (N:) in H_2_TPP, MgTPP, ZnTPP, CuTPP to aminic nitrogen (N–H) in H_2_TPP: (a) TD-DFT BHandHLYP spectral simulations with implicit solvation for the TPP series (black) and their phenyl-free P equivalents (gray). (b) Comparison of the calculated, experimental and negative Kohn–Sham orbital energy shifts.

The detailed assignment of the experimental features is based on the DFT calculations being shown in Fig. 3. As explained above, the BLYP functional is expected to yield reasonable results for the delocalized π* states of the electron-rich porphyrin macrocycle. The comparison of TD-DFT and TP-DFT calculations allows to assess the role of both configuration interactions (CI) and relaxation effects. For investigating the metal–ligand interactions and substituent effects in aqueous solution we refer to Fig. 4(a), where the TD-DFT BHandHLYP spectrum simulations are shown for all investigated TPPs and their phenyl-free P analogs.

### 3.1 Spectral interpretation

The first peak in all experimental spectra (397.9–400.1 eV, blue in Fig. 2a) is the 1e_g_(π*) resonance. The vacant orbitals of the underlying transition are the Gouterman LUMO and LUMO+1, which also participate in the UV/VIS transitions (see Fig. 1). There is general consensus in literature about the assignment of this feature,^34,38–43^ which is not the case for the subsequent resonances.

The narrow 1e_g_ shoulder (399.5 eV, orange), that is only observed for a Cu^2+^ center, results from the half occupied Cu 3d_*x*^2^−*y*^2^_ orbital which mixes with the porphyrin σ-type b_1g_ orbital in *D*_4h_ symmetry. Early solid state measurements of copper porphyrins^39,40,44^ have not been able to resolve this feature. Mangione *et al.*^43^ assigned the b_1g_ resonance to an 1e_g_ shoulder to lower energies – similar to the one which we observe for all samples around 398 eV. Since radiation damage can be excluded for the setup used (see section 2.1) and this shoulder is also visible for the closed-shell ZnTCPP we attribute this feature to thermal geometric distortions. For CuTCPP we interpret the 399.5 eV shoulder on the opposite side of the 1e_g_ peak as σ* resonance. This relative position is in agreement with linear dichroism measurements at the N K-edge of copper phthalocyanine,^45^ explains the existence of a similar shoulder in iron porphyrin in solution,^46^ and is theoretically backed up by our spectrum calculations and the ones by Schmidt *et al.*^47^ for another transition metal TPP.^47^ For CuTPP, the transition appears in both DFT BLYP calculations (see Fig. 3) below the 1e_g_ resonance similar to the calculations done by Mangione *et al.*^43^ The energetic position above the 1e_g_ resonance, being proposed in this work, is based on the benchmarked functionals with a large fraction of HFX as BHandHLYP (see Fig. 4a). This behavior is expected, as hybrid functionals describe charge transfer states more precisely than pure functionals.^10^

The second intense peak in the experimental spectra (400.1–402.0 eV, pink) has been interpreted as b_1g_^47^ or substituent feature^46,48^ in the past. The strong intensity of this peak in all carboxylated metal TPP spectra and the 1e_g_ shoulder, which is only observed for CuTCPP, speak against the former assignment. A substituent transition – in our case phenyl(π*) – is indeed observed in this energy range for TD-DFT based calculations with low or no HFX (cyan in Fig. 3). However, the comparison with experimental octaethylporphyrin^40^ and especially metal P NEXAFS spectra^41,44,49^ (without any outer substituent) make clear that this peak belongs to the π* signature of the porphyrin macrocycle. The second porphyrin peak (P b_2u_ in Fig. 4a) results from the b_2u_(π*) transition. This feature is prominent in all calculations shown. When the TP-DFT and TD-DFT calculations are compared, it can be noted that the b_2u_ resonance is lowered in energy yielding a better agreement with the experimental positions for TP-DFT. At the same time, the 2e_g_ transition is quenched following the general pattern of the core–hole effect to concentrate the excitation intensity on the energetically lower transitions.^50^

The quenching of the 2e_g_ peak by core–hole relaxation is in agreement with results found in literature.^34,44^ Nevertheless, the partial density of unoccupied states has been used to explain the next experimental feature around 401.7 eV (dark green).^38,39,44^ As the 3e_g_ feature has a strong intensity for all calculations with low HFX (see Fig. 3 and ESI†), we assign the third prominent feature to the 3e_g_(π*) transition.

In agreement with previous calculations,^34^ the experimental shoulder around the ionization potential of the metal (M) porphyrins (402.9–403.4 eV, red) can be interpreted as a_2u_ transition. This molecular orbital is formed by hybridization of the porphyrin a_2u_(π*) with M p_*z*_ orbitals.

Above 405 eV, shape resonances can be identified in all experimental spectra (gray). Noticeable is the small blue-shift from MgTCPP to ZnTCPP to CuTCPP indicating a steady contraction of the M–N bond.^51^ This interpretation agrees with the calculated [and experimentally determined^52^] TPP bond length of 2.06 Å for Mg–N, 2.04 Å [2.04 Å] for Zn–N, 2.01 Å [1.98 Å] for Cu–N.

### 3.2 Covalency of the central metal complex

As shown in Fig. 4(b), the experimental 1e_g_ resonance energy increases from N: in H_2_TCPP (397.9 eV) to MgTCPP (398.3 eV) to ZnTCPP (398.5 eV) to CuTCPP (398.7 eV) to N–H in H_2_TCPP (400.1 eV), which is well reproduced by TD-DFT BHandHLYP calculations for the TPP core with implicit solvation. This observation might be surprising, as the 1e_g_ Gouterman orbital is known to be little influenced by the choice of the central moiety.^10,53^ Also differences in the electron–hole interaction between the TPPs and derivatives are expected to be marginal, as the large conjugate system leads to an equally low contribution of the highly delocalized π* orbital at the excited nitrogen atom for all investigated species.^50^

As pointed out by García-Lastra *et al.*,^40^ the energy of the 1e_g_ near-edge resonances mainly depends on the electron density at the nitrogen atoms. This is in line with our calculations as far as the negative Kohn–Sham orbital energies (with 50% HFX) are comparable with ionization potentials. Since a higher electron density at the nitrogen site leads to better screening of the core–hole and thereby lowers the 1e_g_ excitation energy, the negative charge at the nitrogen atoms must decrease in the order: N: in H_2_TPP, MgTPP, ZnTPP, CuTPP, N–H in H_2_TPP (and equally for the TCPPs). Accordingly, the covalency of the N–X (X = Mg, Zn, Cu, H) bond increases. This finding is also in line with the more contracted N–X bond in CuTPP compared to ZnTPP and ZnTPP compared to MgTPP (as discussed above).

The Löwdin orbital populations of the BHandHLYP calculations with implicit solvation reveal an evolution of the complex covalent bond along the series: The N–Mg interaction is mainly characterized by the overlap of magnesium and nitrogen s and p_*x*_/p_*y*_ orbitals (in the porphyrin plane). For the other metal porphyrins, an increased hybridization with metal d_*x*^2^−*y*^2^_ and d_*z*^2^_ atomic orbitals is noticeable. From zinc to copper, π bonding to metal d_*xz*_/d_*yz*_ orbitals gains in importance. That is in line with the established strong mixing of unoccupied metal d and porphyrin π orbitals for open shell transition metals.^53^ In the NEXAFS spectra, the high covalency of the copper complex becomes apparent by the b_1g_ ligand-to-metal charge transfer state, which is missing for the other porphyrins.

Hence, the systematic shift of the 1e_g_ X-ray absorption resonance is a measure of the charge transfer from the porphyrin macrocycle to the metal center. MgTCPP is the most ionic species with an electron density at the nitrogen sites close to the iminic nitrogen atoms in H_2_TCPP. By orbital re-hybridization the ionicity is reduced for ZnTCPP. The strongest metal–porphyrin interactions are observed in CuTCPP where charge delocalization over both the porphyrin macrocycle and the central complex causes a decrease of the nitrogen charge density, which is lower only for N–H in H_2_TCPP.

### 3.3 Covalency to peripheral substituents

As seen in Fig. 4, the central metal atom within the porphyrin ring not only affects the 1e_g_ resonance position but also the energy of the b_2u_ feature. Especially from the comparison of MgTCPP and ZnTCPP (see Fig. 4b) it becomes clear that the interspecies shift of the b_2u_ resonance exceeds the chemical shift of the N 1s core level discussed above.

A shift of the b_2u_ resonance has been experimentally established upon substitution of NiP forming NiTPP.^41^ The observed shift is well reproduced by our TD-DFT calculations with BHandHLYP (see ESI†). Analogously, the calculated spectra of P and TPP are compared for the iminic, magnesium, zinc, copper, and aminic center in Fig. 4(a). It can be seen that the enhanced experimental red-shift of MgTPP is reflected in a larger calculated MgP → MgTPP shift compared to CuP → CuTPP‡‡The b_2u_ feature of ZnP cannot be unambiguously assigned in the TD-DFT BHandHLYP calculation, but seems to be lowered in energy due to strong CI with transitions to mixed zinc atomic and porphyrin π* orbitals..

The experimental evidence for the red-shift upon P substitution with phenyl groups is mirrored in the calculations by CI mixing of the b_2u_ transition with those to covalent porphyrin–phenyl orbitals (see phenyl orbital plot in Fig. 2b). In other words, the rehybridization and mixing of porphyrin and phenyl orbitals and their contribution in the CI expansion continuously decreases from MgTPP to ZnTPP to CuTPP.

The influence of peripheral substituents on the porphyrin electronic structure has rarely been discussed^42,43,53^ or explicitly excluded^40^ in literature. For TPPs this simplification has been justified by the assumption that the phenyl groups are oriented perpendicular (90°) to the porphyrin macrocycle.^34^ However, in practice there is a competition between steric repulsion and π overlap.^54^ Together with geometric distortions induced by the environment this competition leads to phenyl tilts down to 20° on surfaces.^44^ In our calculations, the phenyl tilt amounts to approximately 66°, which is in agreement with X-ray crystallographic data.^55^ In accordance with the assumed increase of phenyl-macrocycle π overlap the calculated phenyl tilt slightly decreases from CuTPP to ZnTPP to MgTPP (see ESI†).

The rise in intensity from CuTCPP to ZnTCPP to MgTCPP around 400 eV (see Fig. 2) can be interpreted as an indicator for increasing covalency leading to the formation of a N(1s) → phenyl(π*) feature in this energy region. This effect would be governed by mixing of the N 2p_*z*_ orbital with the phenyl π* one. However, due to strong vibrational broadening of the intense π* resonances the identification of the phenyl/substituent peak is problematic, nevertheless, its indirect presence can be seen in the shift of the b_2u_ resonance.

Experimental evidence for a change in absorbance around 400 eV in connection to a variation of the substituents is provided by Mangione *et al.*^43^ In their calculations the authors find an increased mixing between porphyrin and substituent orbitals upon fluorine decoration of the phenyl groups in CuTPP opposed to H_2_TPP.

In our series the macrocycle-substituent covalency is increased for a more ionic porphyrin center. That way, the lack of charge transfer with the metal for ZnTPP/ZnTCPP and especially MgTPP/MgTCPP is compensated by an increased charge delocalization onto the substituents. This observation matches with the behaviour of haem, that reduction of the system leads to a global increase of the porphyrin charge density including non-conjugate substituents.^56^

## 4 Conclusions

By the experimental determination and theoretical description of the N K-edge NEXAFS of a series of porphyrins, we have been able to clarify the interpretation of higher π* resonances beyond those of the well-known Gouterman orbitals. We find that even though the electron–hole interaction is of minor importance for relative excitation energies (due to the highly delocalized π system), relaxation effects are crucial for the estimation of excitation intensities. The TP-DFT method is therefore well suited for the description of porphyrin core-excited states. Charge transfer states, however, are only correctly described by hybrid functionals with high HFX, which currently yield theoretically sound results only for TD-DFT.

The experimental and theoretical analysis of systematic interspecies shifts uncovered two trends: The ionicity of the porphyrin center increases from N–H to N–Cu to N–Zn to N–Mg to N:. At the same time the covalency with peripheral substituents increases to compensate for the high charge density at the nitrogen sites.

These findings directly relate to the different functions of metal porphyrins and derivatives. While the ionic character of Mg^2+^macrocycle^2−^ is essential for the charge transfer from negatively charged chlorophyll radicals during photosynthesis, it also gives rise to the low stability of the isolated molecule.^5^ The substitution of magnesium by copper increases the stability of the complex enabling the human utilization beyond its photochemical properties. In the context of technical light-harvesting the observed metal controlled π hybridization of porphyrin and phenyl orbitals are of great interest. Cordones *et al.*^48^ raised the importance of electron localization in the excited state for the efficiency of dye-sensitized solar cells. Zinc porphyrins, which can – based on our results – be seen as a tradeoff between stability and low mixing of metal and porphyrin states, serve as excellent electron acceptors.^48^

Taken together, the investigation of TPP/TCPP orbitals beyond the Gouterman picture revealed the porphyrin center as a regulator for covalency in the entire molecule. In MgTPP/MgTCPP the central N–Mg bond is most ionic so that π hybridization with peripheral substituents is enhanced in compensation. CuTPP/CuTCPP is a counter example with a rather covalent metal–ligand interaction and low π hybridization to the phenyl substituents. Thereby we uncovered the connection between porphyrin constitution and resulting chemical properties which enables the versatile use of porphyrins and derivatives in general.

## Author contributions

R. B.: data curation, investigation, project administration, visualization, writing – original draft; M. F.: resources, investigation; R. H.: investigation; A. P.: supervision; V. V. C.: formal analysis, investigation, supervision; A. F.: conceptualization, funding acquisition, supervision; all: writing – review & editing.

## Conflicts of interest

There are no conflicts to declare.

## Supplementary Material

CP-023-D1CP03944J-s001
