# Supplementary material for: The porphyrin center as a regulator for metal–ligand covalency and π hybridization in the entire molecule
Source: Phys Chem Chem Phys. 2021 Oct 29;23(43):24765–72. doi: 10.1039/d1cp03944j (PMC8579471; doi:10.1039/d1cp03944j)
Supplement: CP-023-D1CP03944J-s001 [file CP-023-D1CP03944J-s001.pdf]

Electronic Supplementary Material (ESI) for Physical Chemistry Chemical Physics.
This journal is © the Owner Societies 2021

Supplementary Information

The Porphyrin Center as a Regulator for Metal-Ligand Covalency and π Hybridization in the Entire Molecule

Robby Büchner,*[a] Mattis Fondell,[b] Robert Haverkamp,[b] Annette Pietzsch,[b] Vinícius Vaz da Cruz,*[b] and Alexander Föhlisch[a,b]

[a] *Institute of Physics and Astronomy, University of Potsdam, Karl-Liebknecht-Str. 24-25, 14476 Potsdam, Germany. Tel: +49 30 806213967; E-mail: rbuechner@uni-potsdam.de,*
[b] *Institute for Methods and Instrumentation for Synchrotron Radiation Research, Helmholtz-Zentrum Berlin für Materialien und Energie, Albert-Einstein-Str. 15, 12489 Berlin, Germany. Tel: +49 30 806214987; E-mail: vinicius.vaz_da_cruz@helmholtz-berlin.de*

UV/VIS spectra

The UV/VIS spectra of all investigated carboxylated tetraphenylporphyrins – more specifically 5,10,15,20-tetrakis(4-carboxyphenyl)porphyrins (TCPPs) – are plotted in Figure S1. They show the characteristic strong Soret (410-430 eV) and weaker Q bands (510-640 eV) common to all porphyrins. The rather narrow lineshape of the former evidences the prevalence of monomers[1].

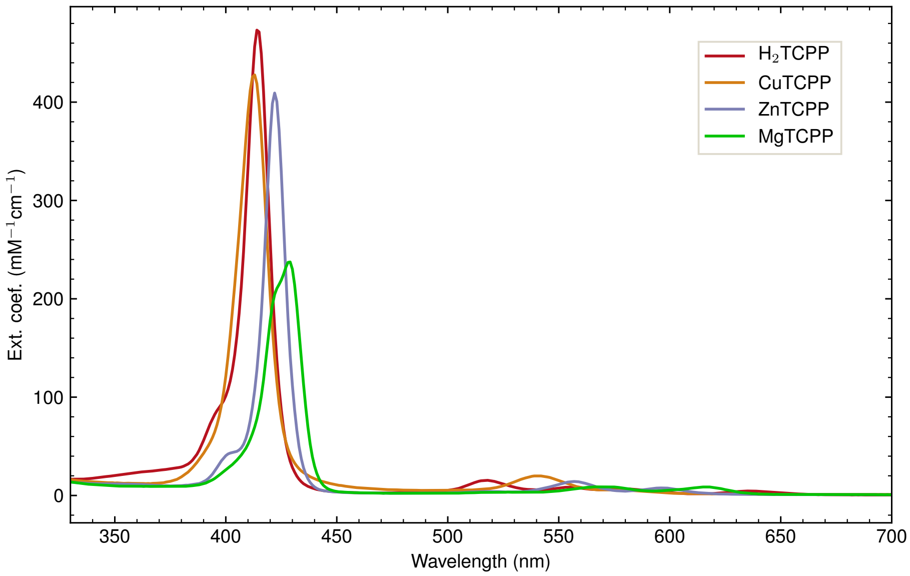

Figure S1 UV/VIS spectra of free base, copper, zinc, and magnesium TCPP in basic aqueous solution.

NEXAFS decomposition

The decomposition of the experimental X-ray absorption spectra of the series of TCPPs at the N K-edge is shown in Figure S2. Voigt profiles with a fixed Lorenzian FWHM of 0.13 eV[2] were fitted for each recognizable resonance in addition to an arctangent step function to model the edge jump.

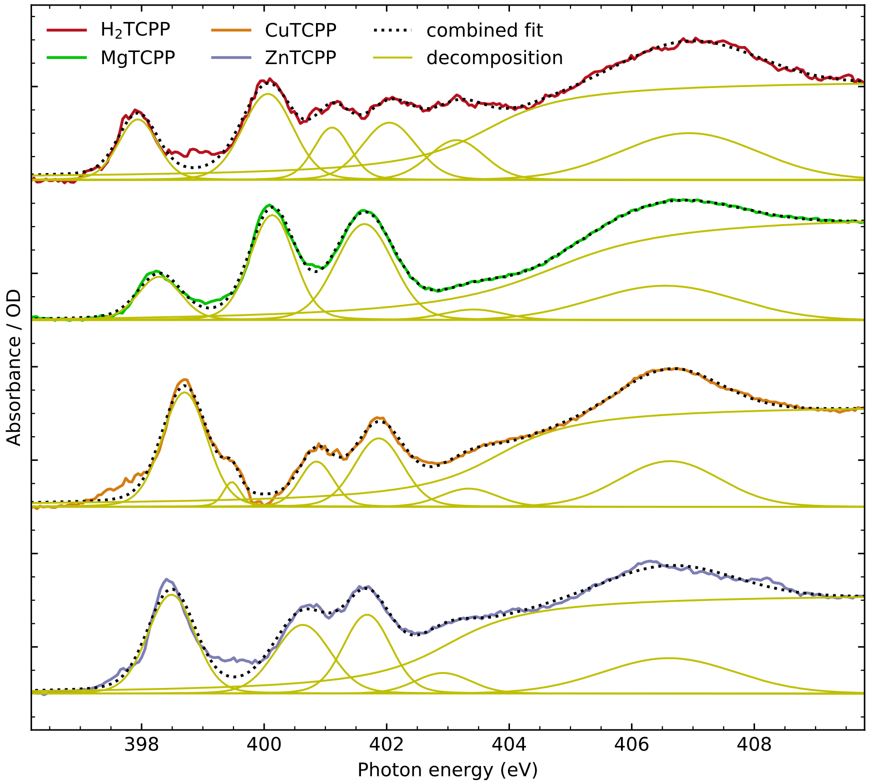

Figure S2 Decomposition of the experimental N K-edge NEXAFS of free base and metal TCPPs by 5-6 Voigt profiles and an arctangent step function.

Molecular geometries

Details of the optimized geometries of the 5,10,15,20-tetraphenylporphyrin (TPP) cores in the gas phase and with implicit solvation (CPCM[3]) are given in Table S1.

Table S1 Calculated TPP N−X bond length (X = H, Mg, Zn, Cu) and phenyl tilt (0° corresponds to a flat arrangement in the porphyrin plane) in the gas phase and with implicit solvation

Molecule	N−X length (gas)	N−X length (CPCM)	phenyl tilt (gas)	phenyl tilt (CPCM)
H_2TPP	2.05 Å	2.06 Å	65.1°	64.1°
MgTPP	2.04 Å	2.04 Å	64.8°	64.5°
ZnTPP	2.01 Å	2.01 Å	66.2°	66.1°
CuTPP	1.01 Å	1.01 Å	66.9°	66.8°

DFT benchmark

In Figure S3, the calculated X-ray absorption spectra of the investigated metal TPP cores are compared. It can be seen that solvation (TD-DFT with CPCM[3] versus gas phase) has a minor effect on the overall spectral shape, but the interspecies shifts are better reproduced with implicit solvation. As discussed in the main article, the inclusion of relaxation by TP-DFT leads to a concentration of the intensities on the energetically lower resonances, which increases the prominence of the experimentally observed features.

If different functionals are compared, it can be seen that the additional feature of CuTPP (b_{1g}) shifts to higher energies with increasing Hartree-Fock exchange (e.g. TD-DFT with CPCM: 398.3 eV for BLYP[4,5], 398.5 eV for B3LYP[6,7], 400.3 eV for BHandHLYP[8]), due to a more accurate description of the metal-ligand interactions. The b_{1g} energy is in qualitative agreement with the experimental data (between the $1e_g$ and b_{2u} peak) for all shown calculations with the BHandHLYP functional and for the cam-B3LYP[9] functional with the transition potential method.

Even though the combination of Hartree-Fock exchange and TP-DFT yields good agreement with the experimental spectra and supports our spectral assignments, this approach is problematic, since the mixing of Kohn-Sham and Hartree-Fock eigenvalues is known to depend strongly on the amount of exchange and core-hole occupation[10].

After all, the experimental shifts are best described by the TD-DFT BHandHLYP calculations, since the transition potential method neglects configuration interactions. As shown in Figure S4, also the shift of the third resonance (b_{2u} around 401.3 eV) upon substitution of NiP is well reproduced by TD-DFT BHandHLYP calculations. The experimental reference has been published by Svirskiy *et al.* [11].

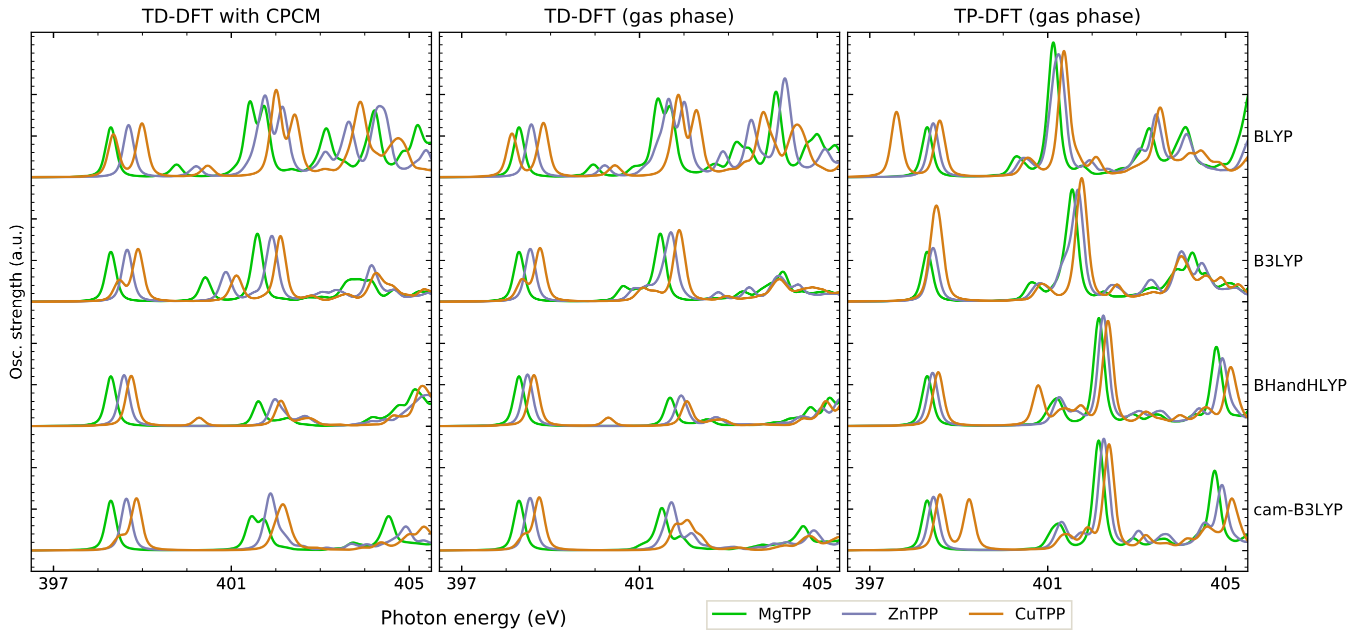

Figure S3 Metal TPP N K-edge NEXAFS calculated by time dependent DFT with and without implicit solvation and by the transition potential method for 4 different functionals (BLYP, B3LYP, BHandHLYP, cam-B3LYP). All transitions were broadened by 0.13 eV[2] (Lorentzian FWHM) and 0.20 eV (Gaussian FWHM) and normalized by the experimental peak height and energy of the first resonance in MgTPP ($1e_g$ at 398.3 eV).

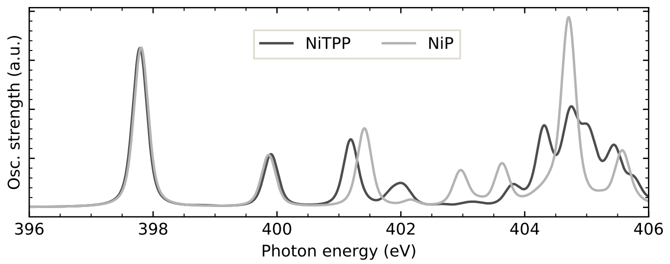

Figure S4 N K-edge NEXAFS of NiP and NiTPP calculated by TD-DFT with the BHandHLYP functional in the gas phase.

References

[1] L. M. Scolaro, M. Castriciano, A. Romeo, S. Patanè, E. Cefalì and M. Allegrini, *J. Phys. Chem. B*, 2002, **106**, 2453–2459.

[2] C. Nicolas and C. Miron, *J. Electron Spectrosc. Relat. Phenom.*, 2012, **185**, 267 – 272.

[3] V. Barone and M. Cossi, *J. Phys. Chem. A*, 1998, **102**, 1995–2001.

[4] A. D. Becke, *Phys. Rev. A*, 1988, **38**, 3098–3100.

[5] C. Lee, W. Yang and R. G. Parr, *Phys. Rev. B*, 1988, **37**, 785–789.

[6] A. D. Becke, *J. Chem. Phys.*, 1993, **98**, 5648–5652.

[7] P. J. Stephens, F. J. Devlin, C. F. Chabalowski and M. J. Frisch, *J. Chem. Phys.*, 1994, **98**, 11623–11627.

[8] A. D. Becke, *J. Chem. Phys.*, 1993, **98**, 1372–1377.

[9] T. Yanai, D. P. Tew and N. C. Handy, *Chem. Phys. Lett.*, 2004, **393**, 51–57.

[10] T. Fransson, I. E. Brumboiu, M. L. Vidal, P. Norman, S. Coriani and A. Dreuw, *J. Chem. Theory Comput.*, 2021, **17**, 1618–1637.

[11] G. I. Svirskiy, A. V. Generalov, A. Y. Klyushin, K. A. Simonov, S. A. Krasnikov, N. A. Vinogradov, A. L. Trigub, Y. V. Zubavichus, A. B. Preobrazhenski and A. S. Vinogradov, *Phys. Solid State*, 2018, **60**, 581–591.
